# Characteristics of the Preoperative and Surgical Findings in Patients With Bladder Dysfunction After Surgery for Spinal Myxopapillary Ependymoma

**DOI:** 10.7759/cureus.71139

**Published:** 2024-10-09

**Authors:** Tomohiro Yamada, Tomohiko Hasegawa, Tomohiko Hasegawa, Go Yoshida, Tomohiro Banno, Hideyuki Arima, Shin Oe, Koichiro Ide, Kenta Kurosu, Yukihiro Matsuyama

**Affiliations:** 1 Orthopedic Surgery, Hamamatsu University School of Medicine, Hamamatsu, JPN; 2 Orthopedic Surgery and Division of Geriatric Musculoskeletal Health, Hamamatsu University School of Medicine, Hamamatsu, JPN

**Keywords:** bladder dysfunction, cauda equina, conus medullaris, gross total resection, myxopapillary ependymoma

## Abstract

*Purpose*: To investigate the association between postoperative bladder dysfunction and preoperative/surgical findings on spinal myxopapillary ependymoma (MPE).

*Methods*: The study included eight patients (five males and three females) with an average age of 56.2 years (range: 21-76 years) who underwent tumor resection between 2011 and 2021. The patient history, magnetic resonance imaging findings, intraoperative findings, surgical methods, and postoperative bladder dysfunction were evaluated. The bladder dysfunction was categorized as mild (frequent urination or retarded micturition) and severe (urinary retention or incontinence).

*Results*: The mean postoperative follow-up was 97.3 (42-160) months. Gross total resection (GTR) was performed in six cases, in three cases by en block resection and three cases by piece-by-piece resection. In GTR cases, an en block resection case with intraoperative adhesion to conus and preoperative normal bladder function presented with postoperative severe bladder dysfunction up to one year. A piece-by-piece resection case with intraoperative adhesion to cauda equina and preoperative mild bladder dysfunction deteriorated to severe dysfunction postoperatively. Subtotal resection (STR) was performed in two cases, which did not show postoperative bladder dysfunction. There was no recurrence of tumor in the all cases.

*Conclusions*: Surgeons should have in mind that in the case with intraoperative adhesion to conus or cauda equina, performing GTR may lead to deteriorated bladder function postoperatively. Careful detachment and gradual intraoperative neuromonitor are necessary to achieve GTR.

## Introduction

Myxopapillary ependymoma (MPE), a rare variant subtype of ependymoma, was first reported in 1932 and is classified as a grade 2 tumor in the latest World Health Organization neurologic tumor classification [1]. MPE frequently occurs in the intra-spinal canal, which is around the conus medullaris (CM) or cauda equina. It can cause local recurrence or disseminated metastasis [2, 3]. MPEs can lead to postoperative bladder dysfunction with surgical manipulation near the spinal cones and are sometimes attached to the cauda equina. The residual tumor can lead to higher tumor recurrence [4], and gross total resection (GTR) has been the gold standard for surgical treatment. However, several studies have reported that GTR is difficult to achieve because of tumor adhesion to the CM or cauda equina [5, 6]. Tumor detachment from surrounding neural tissue may cause postoperative neural dysfunction [7]. Therefore, surgeons often encounter the dilemma of balancing tumor removal and postoperative neurological dysfunction.

Postoperative bladder dysfunction is a unique complication associated with tumor surgery surrounding CM, ranging from a temporary postoperative condition to a long-term condition affecting the quality of life of patients [8]. Several reports have described the intraoperative tumor involvement related to the extent of surgical resection achievable [9]. Postoperative neurological dysfunction may happen in MPEs with GTR [10]*. *However, the characteristics of postoperative bladder dysfunction in patients with MPE and the details of its duration after surgery are unknown. In this series, we aimed to clarify the relationship between the preoperative and surgical findings and postoperative bladder dysfunction and the need to balance extensive surgical resection and postoperative neurological deficits.

## Materials and methods

There are eight pathologically confirmed MPE patients, consecutively treated with surgery at our institute between 2011 and 2021. We obtained the written informed consent from the patients. The Institutional Review Board of our institute approved this study. The medical information of the patients, including clinical data, follow-up duration, magnetic resonance imaging (MRI), surgical details, pathological reports, postoperative adjuvant therapies, and outcomes of follow-ups were collected. All the patients underwent surgical treatment with intraoperative neuromonitoring (IONM). IONM included somatosensory and motor-evoked potentials and free-run electromyography. Based on the surgical records, the degree of resection was divided into GTR and subtotal resection (STR). GTR was defined as no gross residual tumor at the end of the resection and was divided into en block resection and piece-by-piece resection. Walking ability was evaluated using the modified McCormick’s scale [11]. McCormick's clinical and functional spinal cord grading scale was divided into grade I to grade V and was used to assess spinal cord function. We characterized bladder function using three grades (normal, mild, and severe); (1) mild, frequent urination or retarded micturition and (2) severe, urinary retention or incontinence. Follow-up was defined as the duration from the first surgery to the latest outpatient clinic consultation, and recurrence was confirmed based on MRI findings.

## Results

Preoperative clinical characteristics and radiological findings

**Figure 1:**
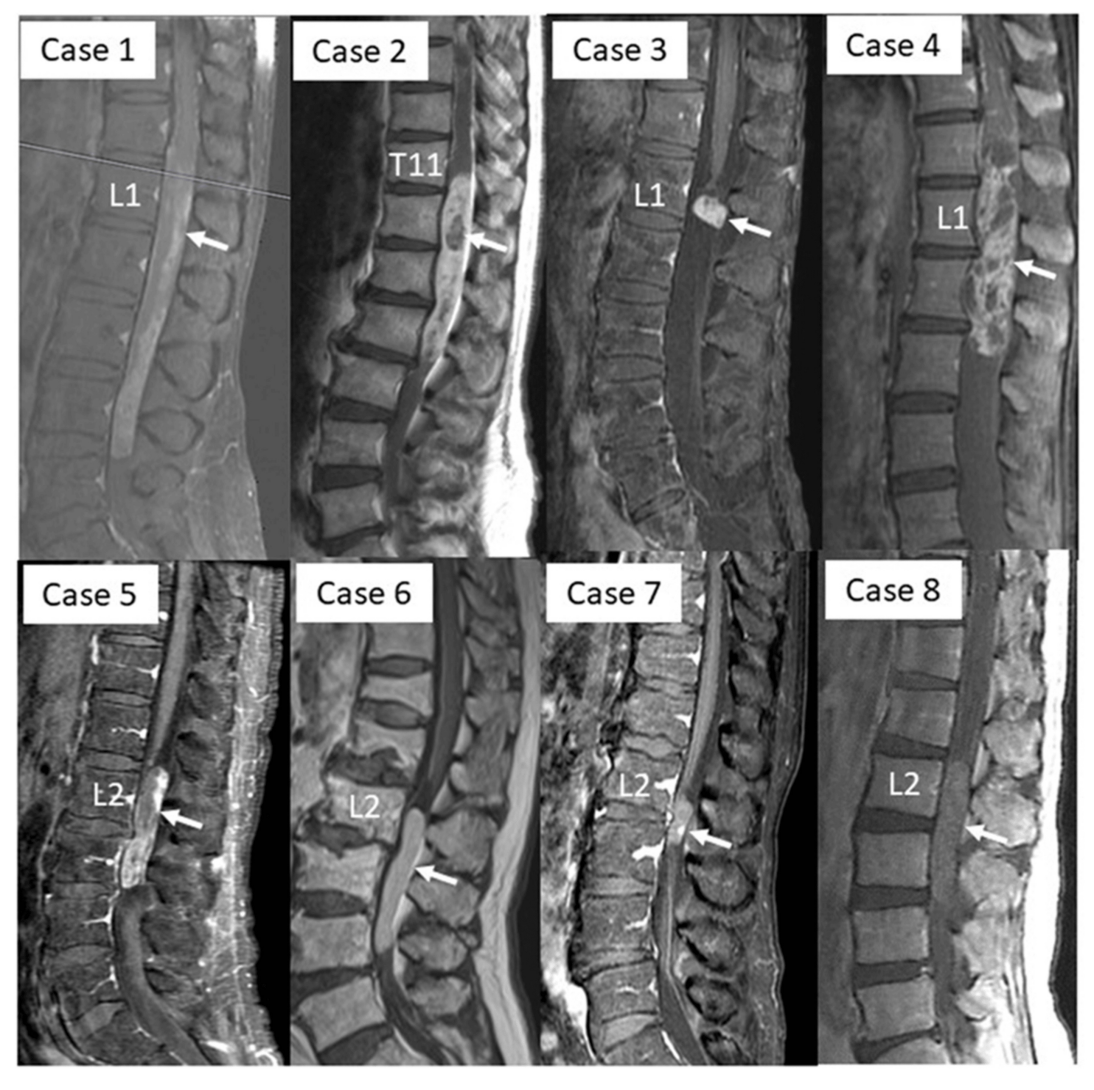
Preoperative enhanced MRI. White arrows indicate the cranial side of tumor location.

The clinical characteristics of the eight patients are presented in Table 1. The study population consisted of five male and three female patients. The average age at the first diagnosis was 56.2 years (range: 21-76 years), and the average duration from symptom onset to surgery was 7.1 months (range: 1-24 years). Tumors were located between the thoracolumbar and lumbar areas. The preoperative symptoms included back and buttock pain (63%), lower muscle weakness (50%), and bladder dysfunction (50%). The preoperative MRI findings are shown in Table 2. The tumor was enhanced homogeneously with gadolinium in two cases and compression change of the conus medullaris in four cases (Figure 1).

**Table 1:** Patients' background and preoperative symptoms. M: Male; F: female; BBD: bowel bladder dysfunction; + (in the BBD column): mild bladder dysfunction; ++ (in the BBD column): severe bladder dysfunction. +, present. -, not present.

Case	Age/Sex	Duration from symptom (months)	Back/Buttock pain	Muscle weakness	BBD
						1	52/F	7	-	-	-
2	43/M	7	-	+	++
3	64/F	4	+	-	++
4	21/F	24	+	-	-
5	55/M	4	+	-	+
6	76/M	1	+	+	+
7	68/M	3	+	+	-
8	50/M	7	-	+	-

**Table 2:** Preoperative MRI findings. CE-MRI: Contrast-enhanced magnetic resonance imaging; H: homogeneously; IH: in-homogeneously; M: male; F: female; +, present. -, not present.

Case	Age/Sex	Tumor levels	CE-MRI	Compression change of conus
					1	52/F	T11-L5	IH	+
2	43/M	T11-L3	IH	+
3	64/F	L1	H	-
4	21/F	T12-L3	IH	+
5	55/M	L1-4	IH	-
6	76/M	L1-4	H	-
7	68/M	L2/3	H	-
8	50/M	L1-4	H	+

Surgical procedures and findings after gross total resection 

Tables 3, 4 show intraoperative surgical findings and postoperative course and pre- to postoperative modified McCormick scale. GTR was achieved for six cases, of which three underwent total block resection without capsule rapture (cases 3, 5, and 7). For cases 3 and 5, we confirmed the tumor surrounding the capsule without adhesion to the cauda equina or conus medullaris. This enabled us to perform en-block GTR. In both cases, the patients had preoperative bowel bladder dysfunction (BBD), which disappeared after tumor resection. However, the tumor was adhesive to the conus medullaris for case 7, and this patient developed postoperative BBD. The patient needed intermittent self-catheterization after one year. The other patients underwent a piece-by-piece procedure (cases 2, 4, 6), all of whom presented with a tumor with capsular rupture. For case 2, there was no adhesion to the cauda equina or conus medullaris, and the BBD improved after the surgery. On the other hand, the tumor was adhesive to the conus medullaris and cauda equina for cases 4 and 6, respectively. We performed GTR with meticulous detachment from surrounding tissues. However, in case 6, the patient showed residual BBD.

**Table 3:** Intraoperative surgical findings and postoperative course. STR: Subtotal resection; GTR: gross total resection; ART: adjuvant radiation therapy; M: male; F: female; Mib-1 index is defined as the percentage of immunoreactive tumor cells in the evaluated area +, present. -, not present.

Case	Age/Sex	Capsule rapture	Adhesion to cauda equina	Adhesion to conus	Resection	Method	ART	Mib-1 index (%)	Recurrence	Total follow-up (months)
											1	52/F	+	+	+	STR		Yes	1-2	No	160
2	43/M	+	-	-	GTR	Piece by piece	Yes	1-2	No	144
3	64/F	-	-	-	GTR	En bloc	No	< 1	No	120
4	21/F	+	-	+	GTR	Piece by piece	Yes	4.4	No	120
5	55/M	-	-	-	GTR	En bloc	No	1-2	No	72
6	76/M	+	+	-	GTR	Piece by piece	No	< 1	No	70
7	68/M	-	-	+	GTR	En bloc	No	1-2	No	50
8	50/M	+	-	+	STR		Yes	< 1	No	40

**Table 4:** Pre- and post-operative bladder dysfunction. GTR: Gross total resection; STR: subtotal resection; +: mild dysfunction, ++: severe dysfunction; M: male; F: female; pre-op: pre-operative; post-op: post-operative

Case	Age/Sex	Resection	Bladder dysfunction				Total follow-up (months)
			Pre-op	Post-op at 1 month	Post-op at 1 year	At final follow-up	
1	52/F	GTR	-	-	-	-	160
2	43/M	GTR	++	+	-	-	144
3	64/F	GTR	++	-	-	-	120
4	21/F	GTR	-	+	-	-	120
5	55/M	GTR	+	-	-	-	72
6	76/M	GTR	+	++	++	++	70
7	68/M	GTR	-	++	++	-	50
8	50/M	STR	-	+	-	-	40

Surgical procedures and findings of subtotal resection 

Subtotal resection (STR) was achieved for two cases as the boundary between the tumor and conus medullaris was unclear (cases 1 and 8). We performed tumor resection with enough surface margin to prevent invading the neural elements. In case 1, the patient showed no neurological deterioration including lower extremity and bowel and bladder functions. In case 2, the patient showed temporary bowel and bladder dysfunction.

Postoperative therapy course

Postoperative adjuvant radiotherapy was performed in four cases, all of whom had intraoperative capsule rupture or underwent STR (cases 1, 2, 4, and 6). The pathological features were consistent with myxopapillary ependymoma in all the cases; they included perivascular pseudorosettes and ependymoma cells. Immunohistochemistry with an anti-MIB-I antibody showed that the average level of expression of the ki-67 labeling index was 2.5% (range: 1%-4.4%) during the surgery (Table 3). The GTR cases showed no recurrence, and the tumor size did not increase for the STR case during the follow-up.

Until the end of the follow-up, the postoperative neurological function evaluated using the McCormick grade was not significantly altered for any of the eight cases (Table 5). During the final follow-up, there were five cases of grade I and one case each of grade II, grade III, and grade IV neurological deficits. Two patients who had not shown BBD preoperatively had urinary dysfunction postoperatively; one patient underwent self-catheterization for over a year. The urinary dysfunction had resolved at the final follow-up for both patients.

**Table 5:** Pre- and post-operative modified McCormick scale. GTR: Gross total resection; STR: subtotal resection; M: male; F: female; pre-op: preoperative; post-op: postoperative

Case	Age/Sex	Resection	McCormick scale			Total follow-up (months)
			Pre-op	Post-op at 1 month	At final follow-up	
1	52/F	GTR	Ⅰ	Ⅰ	Ⅰ	160
2	43/M	GTR	Ⅲ	Ⅲ	Ⅲ	144
3	64/F	GTR	Ⅰ	Ⅰ	Ⅰ	120
4	21/F	GTR	Ⅰ	Ⅱ	Ⅰ	120
5	55/M	GTR	Ⅰ	Ⅰ	Ⅰ	72
6	76/M	GTR	Ⅳ	Ⅳ	Ⅳ	70
7	68/M	GTR	Ⅰ	Ⅰ	Ⅰ	50
8	50/M	STR	Ⅱ	Ⅳ	Ⅱ	40

Representative case (Case 4)

**Figure 2:**
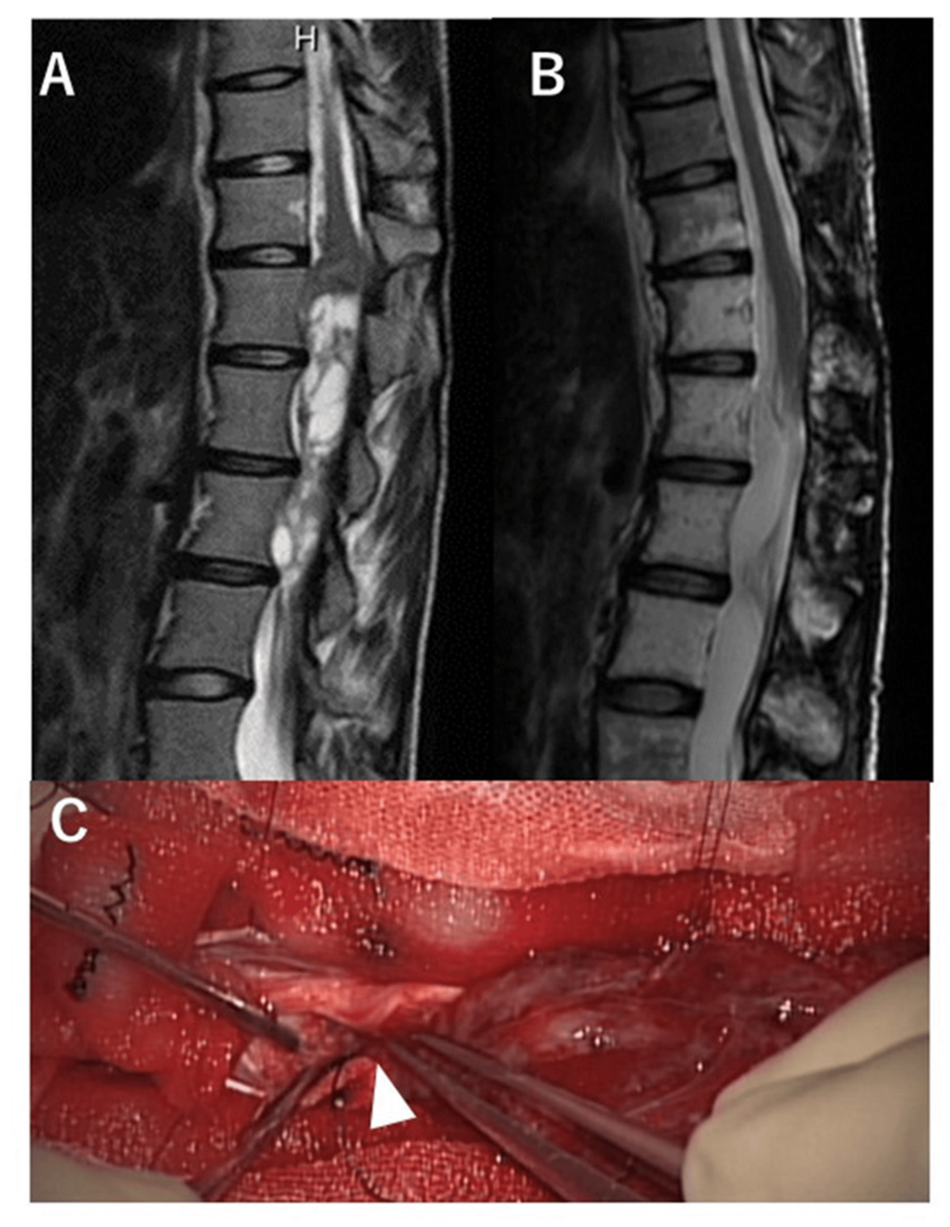
Pre- and postoperative MRI, intraoperative tumor finding. (A) A 21-year-old woman with an intradural tumor around the conus medullaris was shown in a preoperative sagittal T2-weighed MRI. (B) Seven years after postoperative sagittal T2-weighed MRI showed no tumor recurrence. (C) Intraoperative tumor findings showed that the tumor capsule had already ruptured and adhered to the conus medullaris.

**Figure 3:**
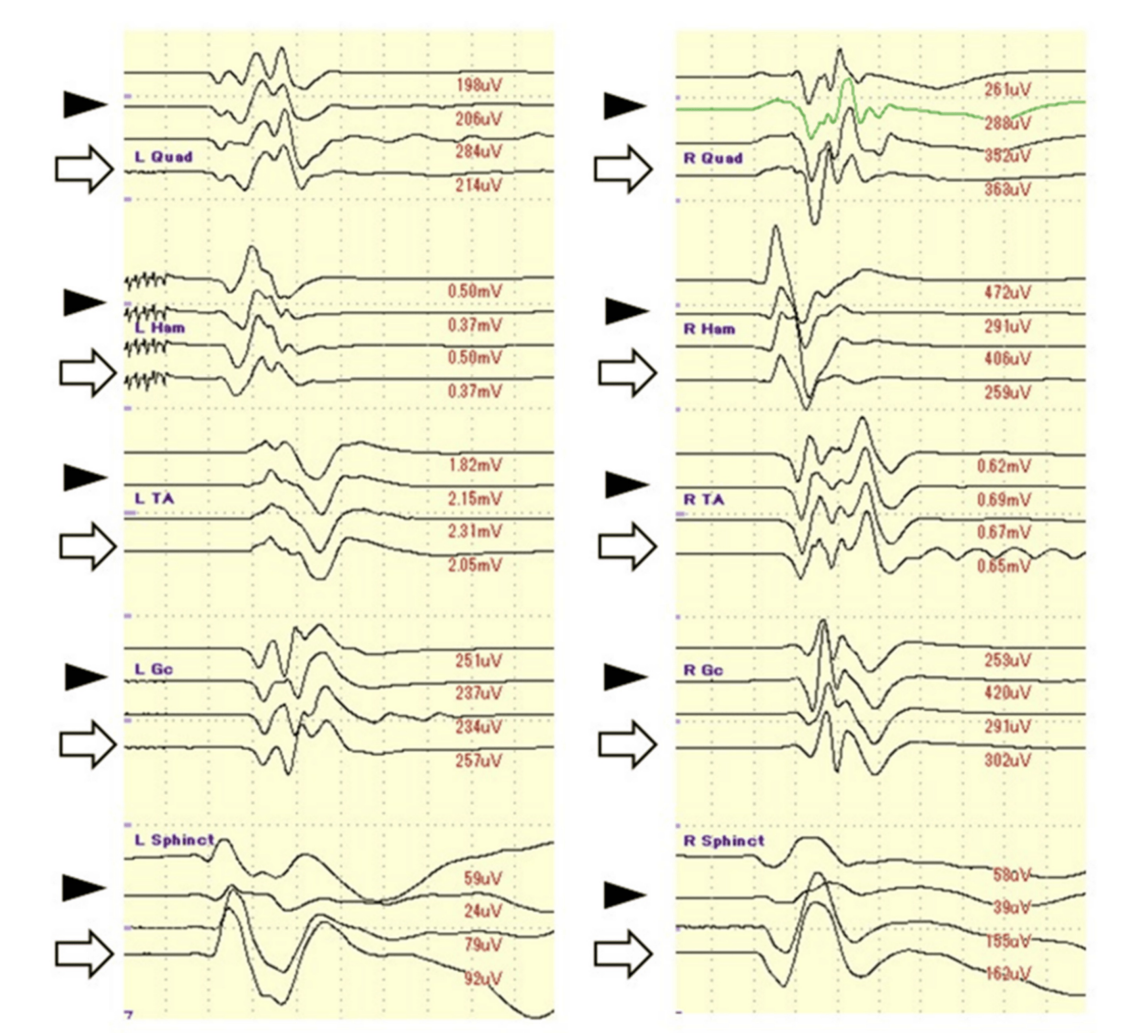
Intraoperative neuromonitoring. During surgery, acceptable baseline waveforms were obtained using transcranial muscle action potential (Tc-MsEP) (open arrow). While detaching the tumor from the conus medullaris, the waveform deteriorated in the right external anal sphincter (EAS). Finally, the EAS waveform decreased to 58 µV (36.0% of the control) on the right and 59 µV (64.1% of the control) on the left.

A 21-year-old female presented to our department with left buttock pain that had persisted for two years. A doctor previously identified a spinal cord tumor from an MRI investigation and referred her to our department. Neurological examination revealed hypoesthesia of the left toe and decreased bilateral Achilles tendon reflexes. MRI revealed an intradural spinal cord tumor at the T12 to L3 level (Figure 2A), and a recapping laminoplasty was performed. Intraoperatively, it was found that the tumor capsule had partially ruptured and adhered to the CM (Figure 2C). The right external anal sphincter motor-evoked potential (EAS-MEP) deteriorated during the detachment of the tumor from the surrounding neural tissue relative to the control amplitude (Figure 3). The right EAS-MEP did not recover after the final stimulation, even after a meticulous detachment. The postoperative urinary dysfunction deteriorated and took the patient six months to recover normal bladder function. After eight years of follow-up, no recurrence of the tumor was observed (Figure 2B).

## Discussion

In our case series, in cases with specific intraoperative findings, including capsule rupture and adhesion to cauda equina and conus medullaris, for whom GTR was performed resulted in postoperative bladder dysfunction (cases 2, 4, 6, and 7). For case 7, capsule rupture was not observed, but the tumor was adhesive to the conus medullaris. After performing GTR, the patient developed bladder dysfunction until one year in the post-operative period.

The extent of surgical resection of MPE is associated with the involvement of the surrounding tissue, such as the CM and cauda equina, and tumor encapsulation [3, 12]. A tumor involving the conus was associated with intraoperative findings and postoperative clinical symptoms. Al-Habib et al. first reported the usefulness of evaluating preoperative tumor involvement in planning surgical treatment [9], and the challenges in achieving GTR for cases with tumor involvement. 

Surgical treatment such as GTR has been the standard therapy because MPE is identified with local recurrence and dissemination in rare cases [13, 14]. GTR cases had significantly longer progression-free survival (PFS) than STR cases [15]. Feldman et al. reported that the recurrence rate after GTR (15.5%) was lower than that after STR (32.6%) irrespective of whether postoperative radiation therapy was referred or not [4]. On the other hand, the interpretation differs from that of Chao et al. who argued that STR would be acceptable for cases with intraoperative tumor adhesion that could induce postoperative neurological deficits [16]. GTR has often been difficult to achieve for cases with tumor involvement in CM [9]. Furthermore, prognosis has not been associated with the surgical procedure, irrespective of whether GTR was performed or not [17].

Tumor adhesion or capsule rupture often makes it challenging to achieve GTR in patients with MPE because of bleeding and neurological dysfunction that may occur during tumor manipulation [18]. Tumor morphology and location in relation to conus medullaris allowed us to anticipate the intraoperative situation and plan for each patient. Helal et al. reported that patients with MPE near the conus were more likely to have postoperative urinary dysfunction [19]. Our study revealed that postoperative bladder dysfunction occurred in cases with tumor adhesion to conus medullaris or cauda equina (Cases 4, 6, 7, and 8). Therefore, STR may be performed to prevent surrounding neural tissue damage in such cases with adhesion to cauda equina or conus medullaris. Actually, our representative case, tumor adhesion to conus medullaris, showed the bladder dysfunction only after performing GTR. STR cases (cases 1 and 8) in our series did not develop a severe postoperative bladder dysfunction.

After performing transcranial muscle-action potentials (Tc-MEPs) for all the cases, GTR was achieved in six cases (75%). Intraoperative monitoring of the EAS was first reported in the 1970s, and monitoring of Tc-MEPs from EAS is now widely performed during spinal surgery to prevent intraoperative neurological deficits [20]. Tc-MEPs provide an assessment of the functional integrity of motor pathways in real time, helping to prevent neurological damage. Kobayashi et al. reported that the amplitude of Tc-MEP-EAS decreased up to 30% of the control waveform, which would be helpful for the prediction of postoperative BBD [21, 22]. For the two cases presented in our study, the EAS-MEP amplitude decreased to approximately 25% of the control wave after detaching the tumor from the conus medullaris (cases 4 and 7), and the postoperative bladder dysfunction was prolonged to six to 12 months. Other channels evaluating the lower extremities, compared with the control waveform, did not show deterioration (Figure 3). This may be explained by the fact that the adhesion was located in the CM, the dysfunction of which was due to saddle anesthesia and BBD without lower muscle weakness [23].

This study had several limitations. First, MPE is a rare tumor, and the cases presented are limited. Second, the follow-up durations were relatively short; the cumulative recurrence rate was high [24]. Third, we did not perform an objective assessment of the bladder function, such as the urodynamic study. We believe that this study contributes to clinical practice by providing insights into optimizing the surgical methods for MPE.

## Conclusions

MPE is a rare type of benign ependymoma with an excellent overall prognosis. However, the adhesion of the tumor to the neural tissue can affect the surgical outcome and partial excision results. To achieve GTR, the preoperative evaluation of the morphological changes of the CM and intraoperative IONM is necessary. The study emphasizes the necessity for meticulous debridement maneuvers and gradual nerve observation during tumor removal.
